# Combined effectiveness of anthelmintic chemotherapy and WASH among HIV-infected adults

**DOI:** 10.1371/journal.pntd.0005955

**Published:** 2018-01-18

**Authors:** Arianna R. Means, Lisette van Lieshout, Eric Brienen, Krista Yuhas, James P. Hughes, Paul Ndungu, Benson Singa, Judd L. Walson

**Affiliations:** 1 Department of Global Health, University of Washington, Seattle, WA, United States; 2 DeWorm3, Natural History Museum, London, United Kingdom; 3 Department of Parasitology, Leiden University Medical Center, Leiden, Netherlands; 4 Department of Biostatistics, University of Washington, Seattle, WA, United States; 5 Centre for Clinical Research, Kenya Medical Research Institute (KEMRI), Nairobi, Kenya; 6 Departments of Medicine, Pediatrics, and Epidemiology, University of Washington, Seattle, WA, United States of America; Baylor College of Medicine, UNITED STATES

## Abstract

**Introduction:**

Current global helminth control guidelines focus on regular deworming of targeted populations for morbidity control. However, water, sanitation, and hygiene (WASH) interventions may also be important for reducing helminth transmission. We evaluated the impact of different potential helminth protective packages on infection prevalence, including repeated treatment with albendazole and praziquantel with and without WASH access.

**Methodology/Principal findings:**

We conducted a cohort study nested within a randomized trial of empiric deworming of HIV-infected adults in Kenya. Helminth infections and infection intensity were diagnosed using semi-quantitative real-time PCR. We conducted a manual forward stepwise model building approach to identify if there are packages of interventions that may be protective against an STH infection of any species (combined outcome) and each helminth species individually. We conducted secondary analyses using the same approach only amongst individuals with no anthelmintis exposure. We used interaction terms to test for potential intervention synergy. Approximately 22% of the 701 stool samples provided were helminth-infected, most of which were of low to moderate intensity. The odds of infection with any STH species were lower for individuals who were treated with albendazole (aOR:0.11, 95%CI: 0.05, 0.20, p<0.001), adjusting for age and sex. Although most WASH conditions demonstrated minimal additional benefit in reducing the probability of infection with any STH species, access to safe flooring did appear to offer some additional protection (aOR:0.34, 95%CI: 0.20, 0.56, p<0.001). For schistosomiasis, only treatment with praziquantel was protective (aOR:0.30 95%CI: 0.14, 0.60, p = 0.001). Amongst individuals who were not treated with albendazole or praziquantel, the most protective intervention package to reduce probability of STH infections included safe flooring (aOR:0.34, 95%CI: 0.20, 0.59, p<0.001) and latrine access (aOR:0.59, 95%CI: 0.35, 0.99, p = 0.05). Across all species, there was no evidence of synergy or antagonism between anthelmintic chemotherapy with albendazole or praziquantel and WASH resources.

**Conclusions/Significance:**

Deworming is effective in reducing the probability of helminth infections amongst HIV-infected adults. With the exception of safe flooring, WASH offers minimal additional benefit. However, WASH does appear to significantly reduce infection prevalence in adults who are not treated with chemotherapy.

**Trial registration:**

ClinicalTrials.gov, NCT00507221.

## Author summary

More than 2 billion people are infected with soil transmitted helminthiasis (STH) and schistosomiasis globally. In addition to deworming with albendazole and praziquantel, water, sanitation, and hygiene (WASH) may be needed to reduce the risk of helminth reinfection and ultimately to break transmission. However evidence on the effectiveness of integrated deworming and WASH programs are mixed. Using data from a randomized controlled trial of deworming among HIV-infected adults in Kenya, we evaluated the associations between different potential packages of deworming and WASH interventions with helminth prevalence. We also aimed to understand if there was a benefit to accessing both WASH and deworming (i.e. synergy). Our findings indicate that deworming treatments were effective in reducing the probability of infection with most helminth species. Of the WASH conditions assessed, safe flooring and latrine access significantly reduced the probability of helminth infections among adults with access to treatment. However WASH may play a more prominent protective role among individuals without access to deworming treatments. These findings also suggest minimal synergistic benefit of coordinated anthelmintic chemotherapy and WASH in this population. These findings have important implications for adult populations who are currently not targeted by deworming campaigns.

## Introduction

The neglected tropical diseases (NTDs) are a group of parasitic and bacterial infections that are associated with substantial global disease and disability, particularly in low income communities. Among the NTDs, helminth infections including schistosomiasis and the soil transmitted helminthiasis (STH) are highly prevalent, infecting over two billion people [1]. Mass drug administration (MDA), or the presumptive treatment of all at risk individuals with preventive chemotherapy, is used to control helminth associated morbidity in endemic areas. In 2001 the World Health Organization (WHO) endorsed MDA as the recommended strategy for controlling STH and schistosomiasis, with a goal of reaching at least 75% of at risk populations and up to 100% of school-aged children [2,3].

The effectiveness of MDA as a control strategy is influenced by the treatment coverage of affected populations, the prevalence and intensity of different helminth species, drug susceptibility and socio-behavioral patterns that influence infection and re-infection rates [4]. In addition, while MDA reduces morbidity by decreasing parasite burden, treatment offers only temporary benefit, as individuals remain at risk of reinfection following treatment. Other strategies, including water, sanitation, and hygiene (WASH) interventions may be needed to reduce the risk of reinfection and ultimately to break transmission of schistosomiasis and STH [5–7]. The presence or absence of WASH, in addition to other social and economic factors, may influence the success of helminth control programs as the intensity of transmission (as estimated by the reproductive number, Ro) is influenced by the survival of free living helminth stages in the surrounding environment [8].

Recent modeling efforts highlight the potential importance of WASH in controlling helminth infections, particularly in high prevalence areas [9,10]. Data from a meta-analysis of water and hygiene protective measures suggest that drinking treated water, soap use, and wearing shoes were associated with approximately 45–60% lower odds of infection [11]. Access to and utilization of sanitation facilities is also associated with significant reductions in the prevalence of all three major STH infections; *A*. *lumbricoides* (OR: 0.54, 95% CI: 0.43–0.69), *T*. *trichiura* (OR: 0.58, 95% CI: 0.45–0.75), and hookworm species (OR: 0.60, 95% CI: 0.48–0.75) [12].

Despite these associations, data from randomized trials evaluating the impact of WASH interventions have demonstrated minimal or no benefit in reducing STH prevalence or intensity in children [13,14]. However, evaluations of the impact of school-based deworming programs indicate that treatment programs have a larger impact on reducing helminth prevalence in areas with improved sanitation facilities, as compared to areas with unimproved or no sanitation [15]. Integrating WASH and MDA strategies may be a promising approach to eliminating disease transmission, however data are limited [16,17]. One cluster-randomized trial in Kenya found a 44% reduction in the odds of *A*. *lumbricoides* infection (but not in other STH species) among children receiving both WASH and MDA, as compared to MDA alone [18]. However, another randomized participatory hygiene and sanitation behavior change intervention trial found no significant difference in helminth infection between the group receiving both WASH and deworming, as compared those receiving deworming alone [19].

Using data from a randomized controlled trial of deworming among HIV infected adults in Kenya, we evaluated associations between WASH access and utilization and helminth prevalence following repeated rounds of treatment with albendazole and praziquantel.

## Methods

### Study design and population

We conducted a retrospective cohort study nested within the Helminth Eradication to delay ART Trial (HEAT) [20]. HEAT was a randomized trial of single-dose albendazole (400 mg) provided every 3 months plus single dose praziquantel (25 mg/kg) provided annually for 2 years to HIV-infected adults. Participants were recruited from HIV clinics at three sites in Kenya (Kisii Provincial Hospital, Kisumu District Hospital, and Kilifi District Hospital) between February 6, 2008, and June 28, 2010. Individuals were eligible for inclusion if they were 18 years of age or older, were HIV seropositive, were not pregnant at the time of enrollment, and did not meet current criteria for ART initiation (on the basis of documented WHO disease stage and CD4+ cell count within the previous 3 months and a clinical assessment at enrollment). Participants were followed every 3 months for 24 months after enrollment.

A single stool sample was obtained from participants after 24 months of follow up. All stool samples were initially examined with a combination of direct microscopy techniques, Kato-Katz and formol-ether concentration by trained laboratory technologists at each of the study sites. Stool aliquots of approximately 1 gram were placed in cryotubes and frozen at −80°C for subsequent DNA extraction and examination at the Leiden University Medical Center (the Netherlands). For this analysis, intensity of helminth infections was assessed by PCR only.

Following a bead-beating step, DNA was isolated using DNeasy 96 Blood & Tissue Kit spin columns in accordance with the manufacturer’s instructions (Qiagen, Hilden, Germany) [21,22]. Phocin herpes virus-1 (PhHV-1) was added to the lysis buffer in each sample as an internal control and virus-specific primers and detecting probe were included in each reaction mixture. Two different multiplex real-time PCR detection panels were used for parasite specific DNA detection of STH and *Schistosoma*: (i) *Ancylostoma spp (*i.e. detecting both *A*. *duodenale* and *A*. *ceylanicum*), *Necator americanus*, *Ascaris lumbricoides*, and *Strongyloides stercoralis*, and (ii) *Schistosoma spp (*i.e. detecting both *S*. *mansoni* and *S*. *haematobium* DNA without differentiation*)* and *Trichuris trichiura* [21]. For the DNA isolation and setup of the PCR reactions a custom-made Hamilton robot platform was used, while amplification, detection and analysis were performed using the CFX real-time detection system (Bio-Rad Laboratories, USA). Negative and positive control samples for each parasite species were included in each PCR run. Fifty PCR amplification cycles were run per sample, with the output expressed as a cycle threshold (Ct). Ct reflects the amplification cycle in which the level of fluorescent signal exceeds background fluorescence and thus species-specific DNA loads in stool samples. For each parasite-specific target, DNA loads were arbitrarily categorized into the following intensity groups, according to previous publications: low (35≤Ct<50), moderate (30≤Ct<35) and high (Ct<30) [23].

In addition to deworming history, four WASH conditions were assessed; access to safe water via access to piped water, purchasing of purified water or treating water independently (filtration, chlorination, boiling, etc), access to a flush latrine or pit latrine in a house or on compound premises, consistent hand washing after using the latrine, and residence in a household in which the floors of the house are made of cement, iron, stone, or timber (i.e. not earthen). Latrines were not identified by improved or unimproved status. Access to these protective WASH conditions were ascertained during longitudinal HEAT surveys. If any of the responses regarding access to WASH resources varied over follow-up, we utilized a participant’s response from the end of the study (when stool samples were collected), or from the preceding three months if not available at study conclusion. Study teams were able to validate reported access to latrines and safe flooring during household visits. Hand washing and safe water access were self-reported by study participants.

The primary outcome of interest was the detection of any STH species infection after 24 months follow-up. As numerous studies have found differential effects of both WASH and deworming by specific helminth species, secondary outcomes of interest included the detection of specific species of helminths [18]. Intensity of infection was examined but could not be assessed due to a lack of statistical power and precision.

We conducted a manual forward stepwise model building approach to identify the potential package of interventions most protective against a helminth infection of any STH species (combined outcome). Individual sex and age were included as covariates in the forward stepwise model building process due to their hypothesized role as potential confounders in the relationship between WASH access and helminth infection. We did not further adjust for SES as the specific WASH resources of interest lie on the causal pathway between SES and helminth infections and access to these WASH resources is a modifiable characteristic that may influence susceptibility to helminth infections.

Multiple imputation using the chained equations (MICE) method was used to address missing values for four of the exposure variables; water treatment status (22% missing), hand washing status (22% missing), safe latrine status (2% missing), and safe flooring status (2% missing). Among individuals not asked about water treatment and hand washing status during surveys, missingness was assumed to be random. Logistic regression was used to impute missing values with ten imputations. Derived estimates from each imputation were combined using Rubin’s methods [24]. We also performed a sensitivity analysis including only complete cases. Estimates from the complete case analysis did not differ qualitatively from those produced with multiple imputation. Therefore, only the effect estimates, 95% confidence intervals (CIs), and p-values derived through imputation are presented.

We used logistic regression and a cutoff threshold of p≤0.1 on the Wald Test as criteria for graduating a variable into the next stepwise model (i.e. branching), or as criteria for removing a variable from a model (i.e. pruning). We started with univariate models that included each independent variable and the outcome. We branched variables that met the cutoff threshold in univariate models into a single multivariate model. After pruning back any variables that did not meet the cutoff threshold, we introduced interaction terms for all independent variable combinations. The purpose of the interaction terms was to identify any potential synergy associated with access to more than one intervention (i.e. a larger protective effect than would be possible with access to one intervention alone). We pruned back any higher level interaction terms that did not meet the cutoff threshold. We then inspected the lower order interaction terms, and pruned back terms that did not meet the cutoff threshold. The branching and pruning stopped once all remaining terms met the cutoff threshold (i.e. equilibrium). Odds ratios (ORs) and their 95% CIs are reported, with significance determined at the alpha = 0.05 level.

We also utilized the manual forward stepwise model building method to identify the optimal package of protective resources relevant to each helminth species. This process was undertaken separately for *A*. *lumbricoides*, hookworm species (all identified infections were *N*. *americanus)*, *T*. *trichiura*, *S*. *stercoralis* and *Schistosoma spp*. Finally, we evaluated the package of WASH resources most protective against helminth infections in HIV-infected adults without access to anthelmintic treatment (the HEAT placebo group) using the manual forward stepwise model building method described above.

### Ethics statement

The University of Washington (UW) Human Subjects Review Committee and the Kenya Medical Research Institute (KEMRI) Ethical Review Committee approved the HEAT study protocol and the trial was registered (ClinicalTrials.gov, number NCT00507221). Study participants were adults who provided written consent in their preferred language (Kiswahili, Kisii, Luo, Giriama, or English) or, if unable to give written consent, provided oral consent in the presence of a witness and confirmed by thumbprint on HEAT study consent forms. Witnesses also signed the study consent forms.

## Results

Of the 740 individuals who participated in HEAT, stool samples were available from 701 individuals. These individuals were predominately young, 44% were 25–34 years old, female (78%) and socio-economically disadvantaged (42% earned less than 2000 Kenyan shillings per month (approximately $20 U.S. dollars). Individuals enrolled in the HEAT study were relatively immunocompetent; median CD4+ cell count was 517 cells/mm3 (interquartile range, IQR: 430–700 cells/mm3) and median viral load was 4.2 log_10_ copies (IQR: 3.5–4.8 log_10_ copies) (Table 1).

**Table 1:** Participant (N = 701) characteristics (non-imputed).

Characteristic	N	%
Female	548	78.2
Avg. (std. dev.) people per household	4.6	2.3
Age		
18–24	94	13.4
25–34	307	43.8
35–44	194	27.7
45–54	69	9.8
55+	37	5.3
Location		
Kilifi	212	29.2
Kisii	257	35.5
Kisumu	256	35.3
Self-reported monthly income (Kenyan shilling)		
<2000	297	43.7
2000–4999	209	30.7
5000–9999	100	14.7
≥10000	74	10.9
*Missing*	21	3.0
Occupation		
Business/self-employed	217	31.7
None	143	20.9
Farmer	137	20.0
Casual laborer	75	11.0
Business/employed by other	57	8.0
Professional (i.e. teacher/lawyer)	55	8.0
*Missing*	17	2.0
Median (IQR) endpoint CD4+ cell count (cells/mm3)	517	430–700
Median (IQR) endpoint log_10 _copies viral load	4.2	3.5–4.8
Received deworming medications in past 6 months as part of trial intervention	348	49.6
*Missing*	3	0.43
Access to treated or piped water (most recent report)	418	59.6
*Missing*	155	22.1
Access to a latrine	480	68.5
*Missing*	17	2.4
Consistent hand washing (most recent report)	393	56.1
*Missing*	155	22.1
Access to safe flooring (i.e. non earthen floors)	325	46.4
*Missing*	17	2.4

Exactly half (50% (n = 348)) of the participants received albendazole and praziquantel in the 6 months preceding stool collection and half (n = 353) received placebo. Nearly 60% (n = 418) of the participants reported access to safe or treated drinking water and 56% (n = 393) reported consistent handwashing after using the latrine. Latrines were accessible in the homes or on the compounds of 66% (n = 480) of study participants and 46% (n = 325) lived in homes with safe, non-earthen flooring. Some of the participants (n = 59, 8%) had access to deworming treatment and all of the protective WASH resources. Only 6 individuals (0.9%) of the participants reported no protective WASH access of any kind.

In total, 152 helminth infections were detected amongst 137 infected individuals using real-time PCR, including: 21 *A*. *lumbricoides*, 60 *N*. *americanus*, 43 *Schistosoma* spp., 8 *S*. *stercoralis*, and 20 *T*. *trichiura* infections. There were 96 STH infections detected, none of which were *Ancylostoma spp*. (Table 2). When evaluating intensity of infection by qPCR, the majority of hookworm, *A*. *lumbricoides*, and *T*. *trichiura* infections were of moderate infection intensity while the majority of *S*. *stercoralis* and *Schistosoma* infections were of low intensity. *S*. *stercoralis* had the highest percentage of high intensity infections of any of the helminth species (25%), while *T*. *trichiura* had the lowest percentage of high intensity infections (5%) (Table 3).

**Table 2:** Prevalent helminth infections.

Species	n (N = 152)	% of helminth infections	% of total population infected
*Ancylostoma spp*.	0	0%	0%
*Ascaris lumbricoides*	21	14%	3%
*Necator americanus*	60	40%	9%
*Schistosoma spp*.	43	28%	6%
*Strongyloides stercoralis*	8	5%	1%
*Trichuris trichiura*	20	13%	3%

**Table 3:** Intensity of prevalent helminth infections (percent of infections).

Species	High intensity (Ct <30)	Moderate intensity (Ct 30–35)	Low intensity (Ct >35)
*Ancylostoma spp*.	NA	NA	NA
*Ascaris lumbricoides*	10%	67%	23%
*Necator americanus*	13%	45%	42%
*Schistosoma spp*.	14%	40%	47%
*Strongyloides stercoralis*	25%	25%	50%
*Trichuris trichiura*	5%	50%	45%

During the stepwise model building process pruning and branching steps were undertaken to derive the package of potential interventions most protective against any STH infection (combined outcome). Only two of the protective interventions remained in the final model: deworming treatment and safe flooring. The odds of infection with any STH species was lower for individuals who were treated relative to those who were not (aOR: 0.11, 95% CI: 0.05, 0.20, p<0.001). The odds of infections with any STH species was also lower for individuals with access to safe flooring relative to those without (aOR: 0.34, 95% CI: 0.20, 0.56, p<0.001) (Table 4). All interaction terms were pruned out of the model, indicating no statistical synergy (or antagonism) across interventions.

**Table 4:** Optimal protective helminth interventions identified through stepwise model building, by species (adjusted for sex and age)^1^.

Species	Treatment (aOR, 95% CI)	Safe floors (aOR, 95% CI)	Latrine (aOR, 95% CI)	Handwashing (aOR, 95% CI)	Safe water (aOR, 95% CI)
Any STH species^2^ (combined)	0.11 (0.05, 0.20)	0.34 (0.20, 0.56)			
*Ascaris lumbricoides*	0.32 (0.11, 0.88)				
*Necator americanus*	0.03 (0.006, 0.11)	0.27 (0.14, 0.52)	0.51 (0.29, 0.92)		
*Strongyloides stercoralis*					
*Trichuris trichiura*	0.11 (0.02, 0.46)				
*Schistosoma spp*	0.30 (0.14, 0.60)			3.14 (0.83, 11.85)	

^**1**^ Grey boxes indicated that variables did not graduate into the final species-specific model based on p≤0.1 cutoff

^**2**^Any STH species includes *Ancylostoma spp*, *Ascaris lumbricoides*, *Necator americanus*, *Strongyloides stercoralis*, *Trichuris trichiura*

The same process was undertaken to derive the most protective package of interventions for each helminth species individually. Treatment (aOR: 0.03, 95% CI: 0.01, 0.11, p<0.001), safe flooring (aOR: 0.27, 95% CI: 0.14, 0.52, p<0.001) and latrine access (aOR: 0.51, 95% CI: 0.29, 0.92, p = 0.03) were included in the potential *N*. *americanus* intervention package (Table 4). Only treatment was associated with reduced probability of infection with *A*. *lumbricoides* (aOR: 0.32, 95% CI: 0.11, 0.88, p = 0.03), *T*. *trichiura* (aOR: 0.11, 95% CI: 0.02, 0.46, p = 0.003) and *Schistosoma sp*. (aOR: 0.30, 95% CI 0.14, 0.60, p = 0.001). Interestingly, handwashing was associated with an elevated probability of *Schistosoma sp*. infection (aOR: 3.14, 95% CI: 0.83, 11.85, p = 0.09). No interventions were associated with reductions in *S*. *stercoralis* infection.

In the absence of anthelminthic treatment, the most protective package for any STH infection included safe flooring (aOR: 0.34, 95% CI: 0.20, 0.59, p<0.001) and latrine access (aOR: 0.59, 95% CI: 0.35, 0.99, p = 0.05) (Table 5). For the specific outcome of *N*. *americanus*, safe flooring (aOR: 0.25, 95% CI: 0.13, 0.50, p<0.001) and latrine access (aOR: 0.47, 95% CI: 0.26, 0.86, p = 0.01) appeared most protective. For *A*. *lumbricoides*, the most protective package not including anthelmintics contained safe water only (OR: 0.37, 95% CI: 0.12, 1.12, p = 0.08). No intervention was effective at reducing the probability of *T*. *trichiura* infection in the absence of anthelminthics. Consistent handwashing was associated with an increased risk of *Schistosoma sp*. infection in the absence of anthelmintic therapy (OR: 3.62, 95% CI: 0.78, 16.92, p = 0.10). Finally, latrine access appeared to reduce the probability of infection with *S*. *stercoralis* in the absence of treatment (OR: 0.13, 95% CI: 0.01, 1.15, p = 0.07). Across all species, interaction terms were pruned out of the models, indicating no statistical synergy (or antagonism) across interventions.

**Table 5:** Optimal protective helminth interventions in adults without access to treatment identified through stepwise model building, by species (adjusted for sex and age) ^1^.

Species	Safe floors (aOR, 95% CI)	Latrine (aOR, 95% CI)	Handwashing (aOR, 95% CI)	Safe water (aOR, 95% CI)
Any STH species^**2**^ (combined)	0.34 (0.20, 0.59)	0.59 (0.35, 0.99)		
*Ascaris lumbricoides*				0.37 (0.12, 1.12)
*Necator americanus*	0.25 (0.13, 0.50)	0.47 (0.26, 0.86)		
*Strongyloides stercoralis*		0.13 (0.01, 1.15)		
*Trichuris trichiura*				
*Schistosoma spp*			3.62 (0.78, 16.92)	

^**1**^ Grey boxes indicated that variables did not graduate into the final species-specific model based on p≤0.1 cutoff

^**2**^Any STH species includes *Ancylostoma spp*, *Ascaris lumbricoides*, *Necator americanus*, *Strongyloides stercoralis*, *Trichuris trichiura*

We assessed the pairwise correlation between the independent variables in order to understand if multicollinearity was affecting the variance in the models, and thus causing some independents variables to appear statistically insignificant and drop out of the stepwise model building process. Correlation appeared to be mild, ranging from -0.05 (treatment and safe water) to 0.17 (safe flooring and toilet access). We also performed a sensitivity analysis including only complete cases. Estimates from the complete case analysis did not differ qualitatively from those produced with multiple imputation. Therefore, only the effect estimates, 95% CIs, and p-values derived through imputation are presented.

## Discussion

Given recent calls for the elimination of several NTDs by 2020, evaluation of multilateral approaches to morbidity control and interruption of disease transmission through combination interventions including MDA and WASH programs is critically important. In this analysis, both treatment with albendazole and praziquantel and access to some WASH resources demonstrated benefit in reducing the probability of STH and schistosomiasis infections. Despite this, in this study there was no evidence of additional benefit (synergy) associated with access to more than one WASH resource when combined with chemotherapy. However, access to several WASH resources did appear to demonstrate benefit in reducing STH prevalence and intensity in adults who did not receive albendazole or praziquantel.

Treatment with albendazole and praziquantel was effective in preventing all helminth infections with the exception of *S*. *stercoralis*. This is consistent with previous studies documenting high treatment efficacy of albendazole for *A*. *lumbricoides (*cure rate of 88%) and hookworm species (cure rate of 72%), and of praziquantel for schistosomiasis (cure rate of 76%). It is also consistent with the documented poor treatment efficacy of albendazole for *S*. *stercoralis*.[25]. The finding of consistent benefit of albendazole for *T*. *Trichiura* was somewhat surprising given previous data suggesting relatively poor treatment efficacy for this organism *(*28%), and may be due to the fact that individuals in this study were treated with albendazole repeatedly over 24 months [4,26].

In this study, 60% and 69% of participants had access to safe drinking water and latrine facilities, respectively. However, among individuals who received deworming medications, access to safe water did not appear to provide additional benefit in reducing helminth infections and access to latrines only appeared to reduce infection with hookworm species. The apparent contradiction between these findings and understood mechanisms of helminth exposure are echoed in previous studies [14,27], including a three year cluster randomized trial with over 50,000 participants in India that found no association between increased latrine coverage and reductions in helminth infection or intensity [13]. These findings suggest that the additional benefit of reducing exposure in individuals receiving repeated rounds of chemotherapy may be limited. In addition, access to safe water and latrines is not equivalent to consistent or effective uptake of the resources, and cultural norms regarding use of these resources greatly influence helminth exposure.

Despite the inconsistent benefit of WASH demonstrated in this study, reducing exposure through primary transmission routes may be particularly important in populations not targeted by global guideliens for routine deworming, including adults. Amongst adults who did not receive albendazole or praziquantel, the impact of WASH in reducing STH infection was more pronounced. In untreated participants, access to latrines reduced the probability of infection of both hookworm and *S*. *stercoralis* and access to safe water reduced the probability of *A*. *lumbricoides* infection. This finding is consistent with previous evidence suggesting that untreated individuals without access to improved latrines have an increased odds of infection with skin penetrating helminths such as hookworm and *S*. *stercoralis* (combined aOR 3.9, 95% CI: 2.6–5.9) and that unimproved drinking water is associated with increased odds of infection with orally-ingested helminths such as *A*. *lumbricoides and T*. *trichiura* (combined aOR = 2.2; 95% CI: 1.3–3.7) [28].

In many settings, handwashing has also been associated with prevention of intestinal parasitic infections [29–33]. However, predictive models from the data presented here suggest that consistent handwashing may not provide benefit and, in fact, may be associated with an increased probability of *Schistosoma* infection. This may be due to self-reporting bias that misrepresents the true number of people practicing consistent hand washing. Alternatively, this observation may be due to a practice of handwashing with water from *Schistosoma* contaminated water sources. Individuals who live in close proximity to water sources may be more likely to practice handwashing and also more likely to be exposed to infective cercariae. Handwashing with soap is recommended when washing hands with fresh water in endemic areas in order to reduce the infectivity of cercariae, and data regarding individual soap use was not collected in this study [34].

In this analysis, the WASH resource that appeared to have the largest and most consistently protective effect was residence in a household with non-earthen floors. Earthen floor have been shown to be a significant risk factor for hookworm infection, responsible for as much as 86% of all hookworm infections in West Africa [35]. Given that 44% of helminth infections in this cohort were hookworm species (as expected in an adult population), it is likely that safe flooring was particularly important for preventing infection in this group. Although we did not measure shoe wearing practices, consistent use of footwear has also been shown to lower the odds of hookworm infection [18,36].

While biologic rationale would seem to indicate that integration of deworming and WASH interventions would have a synergistic effect on helminth prevalence through dual removal of extant infections and sources of exposure, there was no indication of such synergy in our study. These observations are in contrast to findings from a randomized trial that identified a significant reduction in *A*. *lumbricoides* infections amongst school children who received a school-based WASH program plus albendazole as compared to children who received albendazole alone [18]. Other studies have also found that health education, latrine construction, and deworming significantly reduce STH prevalence [37], and a systematic review concluded that combined sanitation, education, and deworming reduced STH prevalence by more than 60% relative to sanitation and education alone [38]. New efforts are underway to understand the influence of integrated WASH activities and mass deworming of entire communities using a cluster randomized trial design [39].

There are several notable strengths of this analysis. These data were rigorously collected as part of a randomized trial of deworming interventions and highly sensitive molecular diagnostic tools were used to detect helminth infections. Given the poor sensitivity of microscopic techniques in low prevalence areas, including this study site [40], and the fact that 39% of infections in this cohort were low intensity, it is likely that we detected infections that would have gone undetected using standard microscopic methods. However, there were also several limitations associated with this retrospective cohort study. First, we were unable to document true WASH usage, adherence, and behavior patterns and using WASH access as a surrogate for WASH usage may be problematic [41]. In addition, our data do not provide insight into food preparation or shoe wearing practices, which could also influence helminth exposure and infection estimates. Although individuals did report whether they had access to latrines, we did not assess whether or not the latrines were improved or unimproved or if they were well maintained as original HEAT surveys did not capture UNICEF Joint Monitoring Programme indicators. Additionally, it is possible that repeat surveys regarding WASH access affected participant behavior, such that the final survey was not necessarily a representative index of risk throughout follow-up. Also, although the *Schistosoma* PCR targeted both *S*.*mansoni* and *S*. *haematobium*, our ability to detect *S*. *haematobium* DNA in stool may be limited. In the current study we think we have not missed many *S*. *haematobium* infections because urine microscopy revealed less than 1% prevalence. Finally, because the study was conducted in HIV-infected adults, the generalizability of our findings may be limited. However, there is some evidence that HIV infected adults may benefit from deworming and the potential additive role of WASH in this population warrants further investigation [42]. Lastly, if helminth acquired immunities are age-dependent, one might expect these protective resources to have more pronounced influences in younger populations.

### Conclusion

Access to WASH is unequivocally essential, it is a human rights necessity and a hallmark of the Sustainable Development Goals. However evidence supporting the additive benefit of integrated deworming and WASH strategies has been mixed, in part due to the logistical and ethical challenges surrounding the design and implementation of large randomized WASH studies [43]. In this study, anthelmintic treatment was shown to be highly effective in reducing the probability of helminth infections. WASH resources, with the exception of safe flooring and latrine access, did not appear to provide substantial additive benefit to chemotherapy. However, among individuals not receiving chemotherapy, there was clear benefit of WASH in reducing the probability of helminth infections. Given the complexity of integrating multisectorial approaches to disease control [44], researchers and NTD programs should carefully consider if and how pursuing integrated chemotherapeutic and WASH strategies may influence capacity to deliver high quality interventions and achieve targeted outcomes.

## Supporting information

S1 Checklist STROBE checklist. (DOC)

S1 File Study data. (XLS)

## References

[1] WHO (2015) Soil-transmitted helminth infections. World Health Organization. pp. Media Centre.

[2] WHO (2002) Prevention and control of schistosomiasis and soil-transmitted helminthiasis: report of a WHO expert committee. Geneva. 1–57 p. 12592987

[3] WHO (2013) Rolling out and scaling up integrated preventive chemotherapy for selected neglected tropical diseases. Geneva: World Health Organization 161–172 p. 23620908

[4] Keiser J, Utzinger J (2008) Efficacy of current drugs against soil-transmitted helminth infections: systematic review and meta-analysis. Jama 299: 1937–1948. doi: 10.1001/jama.299.16.1937 18430913 10.1001/jama.299.16.1937

[5] Utzinger J, Raso G, Brooker S, De Savigny D, Tanner M, et al (2009) Schistosomiasis and neglected tropical diseases: Towards integrated and sustainable control and a word of caution. Parasitology 136: 1859–1874. doi: 10.1017/S0031182009991600 19906318 10.1017/S0031182009991600 PMC2791839

[6] Utzinger J, Bergquist R, Shu-Hua X, Singer BH, Tanner M (2003) Sustainable schistosomiasis control—the way forward. Lancet 362: 1932–1934. doi: 10.1016/S0140-6736(03)14968-9 14667754 10.1016/S0140-6736(03)14968-9

[7] Hotez P (2006) Helminth Infections: Soil-transmitted Helminth Infections and Schistosomiasis Disease Control Priorities in Developing Countries. 2nd ed: World Bank.

[8] Brooker S, Clements AC, Bundy DA (2006) Global epidemiology, ecology and control of soil-transmitted helminth infections. Adv Parasitol 62: 221–261. doi: 10.1016/S0065-308X(05)62007-6 16647972 10.1016/S0065-308X(05)62007-6 PMC1976253

[9] Coffeng LE, Bakker R, Montresor A, de Vlas SJ (2015) Feasibility of controlling hookworm infection through preventive chemotherapy: a simulation study using the individual-based WORMSIM modelling framework. Parasit Vectors 8: 541 doi: 10.1186/s13071-015-1151-4 26489659 10.1186/s13071-015-1151-4 PMC4618856

[10] Anderson R, Truscott J, Hollingsworth TD (2014) The coverage and frequency of mass drug administration required to eliminate persistent transmission of soil-transmitted helminths. Philos Trans R Soc Lond B Biol Sci 369: 20130435 doi: 10.1098/rstb.2013.0435 24821921 10.1098/rstb.2013.0435 PMC4024228

[11] Strunz EC, Addiss DG, Stocks ME, Ogden S, Utzinger J, et al (2014) Water, sanitation, hygiene, and soil-transmitted helminth infection: a systematic review and meta-analysis. PLoS Med 11: e1001620 doi: 10.1371/journal.pmed.1001620 24667810 10.1371/journal.pmed.1001620 PMC3965411

[12] Ziegelbauer K, Speich B, Mäusezahl D, Bos R, Keiser J, et al (2012) Effect of Sanitation on Soil-Transmitted Helminth Infection: Systematic Review and Meta-Analysis. PLoS Med. 10.1371/journal.pmed.1001162 PMC3265535 22291577

[13] Clasen T, Boisson S, Routray P, Torondel B, Bell M, et al (2014) Effectiveness of a rural sanitation programme on diarrhoea, soil-transmitted helminth infection, and child malnutrition in Odisha, India: a cluster-randomised trial. The Lancet Global Health 2. 10.1016/S2214-109X(14)70307-9 25442689

[14] Patil SR, Arnold BF, Salvatore AL, Briceno B, Ganguly S, et al (2014) The effect of India's total sanitation campaign on defecation behaviors and child health in rural Madhya Pradesh: a cluster randomized controlled trial. PLoS Med 11: e1001709 doi: 10.1371/journal.pmed.1001709 25157929 10.1371/journal.pmed.1001709 PMC4144850

[15] Nikolay B, Mwandawiro CS, Kihara JH, Okoyo C, Cano J, et al (2015) Understanding Heterogeneity in the Impact of National Neglected Tropical Disease Control Programmes: Evidence from School-Based Deworming in Kenya. PLoS Negl Trop Dis 9: e0004108 doi: 10.1371/journal.pntd.0004108 26421808 10.1371/journal.pntd.0004108 PMC4589351

[16] Freeman MC, Ogden S, Jacobson J, Abbott D, Addiss DG, et al (2013) Integration of water, sanitation, and hygiene for the prevention and control of neglected tropical diseases: a rationale for inter-sectoral collaboration. PLoS Negl Trop Dis 7: e2439 doi: 10.1371/journal.pntd.0002439 24086781 10.1371/journal.pntd.0002439 PMC3784463

[17] Campbell SJ, Savage GB, Gray DJ, Atkinson JA, Soares Magalhaes RJ, et al (2014) Water, Sanitation, and Hygiene (WASH): a critical component for sustainable soil-transmitted helminth and schistosomiasis control. PLoS Negl Trop Dis 8: e2651 doi: 10.1371/journal.pntd.0002651 24722335 10.1371/journal.pntd.0002651 PMC3983087

[18] Freeman MC, Clasen T, Brooker SJ, Akoko DO, Rheingans R (2013) The impact of a school-based hygiene, water quality and sanitation intervention on soil-transmitted helminth reinfection: a cluster-randomized trial. Am J Trop Med Hyg 89: 875–883. doi: 10.4269/ajtmh.13-0237 24019429 10.4269/ajtmh.13-0237 PMC3820330

[19] Dumba R, Kaddu JB, Wabwire-Mangen F (2013) Design and implementation of participatory hygiene and sanitation transformation (PHAST) as a strategy to control soil-transmitted helminth infections in Luweero, Uganda. Afr Health Sci 13: 512–517. doi: 10.4314/ahs.v13i2.44 24235957 10.4314/ahs.v13i2.44 PMC3824518

[20] Walson J, Singa B, Sangare L, Naulikha J, Piper B, et al (2012) Empiric deworming to delay HIV disease progression in adults with HIV who are ineligible for initiation of antiretroviral treatment (the HEAT study): a multi-site, randomised trial. Lancet Infect Dis 12: 925–932. doi: 10.1016/S1473-3099(12)70207-4 22971323 10.1016/S1473-3099(12)70207-4

[21] Kaisar MM BE, Djuardi Y, Sartono E, Yazdanbakhsh M, Verweij JJ, Supali T, Van Lieshout L (2017) Improved diagnosis of Trichuris trichiura by using a bead-beating procedure on ethanol preserved stool samples prior to DNA isolation and the performance of multiplex real-time PCR for intestinal parasites. Parasitology (IN PRESS). 10.1017/S0031182017000129 PMC5471844 28290266

[22] Kaisar MMM, Brienen EAT, Djuardi Y, Sartono E, Yazdanbakhsh M, et al (2017) Improved diagnosis of Trichuris trichiura by using a bead-beating procedure on ethanol preserved stool samples prior to DNA isolation and the performance of multiplex real-time PCR for intestinal parasites. Parasitology 144: 965–974. doi: 10.1017/S0031182017000129 28290266 10.1017/S0031182017000129 PMC5471844

[23] Pillay P, Taylor M, Zulu SG, Gundersen SG, Verweij JJ, et al (2014) Real-time polymerase chain reaction for detection of Schistosoma DNA in small-volume urine samples reflects focal distribution of urogenital Schistosomiasis in primary school girls in KwaZulu Natal, South Africa. Am J Trop Med Hyg 90: 546–552. doi: 10.4269/ajtmh.13-0406 24470560 10.4269/ajtmh.13-0406 PMC3945702

[24] Rubin D (1987) Multiple imputation for nonresponse in surveys. New York: John Wiley & Sons.

[25] Beknazarova M, Whiley H, Ross K (2016) Advocating for both Environmental and Clinical Approaches to Control Human Strongyloidiasis. Pathogens 5. 10.3390/pathogens5040059 PMC5198159 27706031

[26] Liu R, Dong HF, Guo Y, Zhao QP, Jiang MS (2011) Efficacy of praziquantel and artemisinin derivatives for the treatment and prevention of human schistosomiasis: a systematic review and meta-analysis. Parasit Vectors 4: 201 doi: 10.1186/1756-3305-4-201 22004571 10.1186/1756-3305-4-201 PMC3207908

[27] Campbell SJ, Nery SV, Wardell R, D'Este CA, Gray DJ, et al (2017) Water, Sanitation and Hygiene (WASH) and environmental risk factors for soil-transmitted helminth intensity of infection in Timor-Leste, using real time PCR. PLoS Negl Trop Dis 11: e0005393 doi: 10.1371/journal.pntd.0005393 28346536 10.1371/journal.pntd.0005393 PMC5383321

[28] Echazu A, Bonanno D, Juarez M, Cajal SP, Heredia V, et al (2015) Effect of Poor Access to Water and Sanitation As Risk Factors for Soil-Transmitted Helminth Infection: Selectiveness by the Infective Route. PLoS Negl Trop Dis 9: e0004111 doi: 10.1371/journal.pntd.0004111 26421865 10.1371/journal.pntd.0004111 PMC4589369

[29] Xu LQ, Xiao DH, Zhou CH, Zhang XQ, Lan SG, et al (2001) [On cleanliness of hands in diminution of Ascaris lumbricoides infection in children]. Zhongguo Ji Sheng Chong Xue Yu Ji Sheng Chong Bing Za Zhi 19: 294–297. 12572046

[30] Bieri FA, Gray DJ, Williams GM, Raso G, Li YS, et al (2013) Health-education package to prevent worm infections in Chinese schoolchildren. N Engl J Med 368: 1603–1612. doi: 10.1056/NEJMoa1204885 23614586 10.1056/NEJMoa1204885

[31] Gyorkos TW, Maheu-Giroux M, Blouin B, Casapia M (2013) Impact of health education on soil-transmitted helminth infections in schoolchildren of the Peruvian Amazon: a cluster-randomized controlled trial. PLoS Negl Trop Dis 7: e2397 doi: 10.1371/journal.pntd.0002397 24069469 10.1371/journal.pntd.0002397 PMC3772033

[32] Mahmud MA, Spigt M, Bezabih AM, Pavon IL, Dinant GJ, et al (2015) Efficacy of Handwashing with Soap and Nail Clipping on Intestinal Parasitic Infections in School-Aged Children: A Factorial Cluster Randomized Controlled Trial. PLoS Med 12: e1001837; discussion e1001837. doi: 10.1371/journal.pmed.1001837 26057703 10.1371/journal.pmed.1001837 PMC4461173

[33] Gelaw A, Anagaw B, Nigussie B, Silesh B, Yirga A, et al (2013) Prevalence of intestinal parasitic infections and risk factors among schoolchildren at the University of Gondar Community School, Northwest Ethiopia: a cross-sectional study. BMC Public Health. pp. 304. 10.1186/1471-2458-13-304 PMC3621079 23560704

[34] Grimes JE, Croll D, Harrison WE, Utzinger J, Freeman MC, et al (2015) The roles of water, sanitation and hygiene in reducing schistosomiasis: a review. Parasit Vectors. 10.1186/s13071-015-0766-9 PMC4377019 25884172

[35] Soares Magalhaes RJ, Barnett AG, Clements AC (2011) Geographical analysis of the role of water supply and sanitation in the risk of helminth infections of children in West Africa. Proc Natl Acad Sci U S A 108: 20084–20089. doi: 10.1073/pnas.1106784108 22123948 10.1073/pnas.1106784108 PMC3250163

[36] Alemu A, Atnafu A, Addis Z, Shiferaw Y, Teklu T, et al (2011) Soil transmitted helminths and schistosoma mansoni infections among school children in Zarima town, northwest Ethiopia. BMC Infect Dis 11: 189 doi: 10.1186/1471-2334-11-189 21740589 10.1186/1471-2334-11-189 PMC3142518

[37] Steinmann P, Yap P, Utzinger J, Du ZW, Jiang JY, et al (2014) Control of soil-transmitted helminthiasis in Yunnan province, People's Republic of China: experiences and lessons from a 5-year multi-intervention trial. Acta Trop 141: 271–280. doi: 10.1016/j.actatropica.2014.10.001 25308524 10.1016/j.actatropica.2014.10.001

[38] Asaolu SO, Ofoezie IE (2003) The role of health education and sanitation in the control of helminth infections. Acta Trop 86: 283–294. 12745145 10.1016/s0001-706x(03)00060-3

[39] Nery SV, McCarthy JS, Traub R, Andrews RM, Black J, et al (2015) A cluster-randomised controlled trial integrating a community-based water, sanitation and hygiene programme, with mass distribution of albendazole to reduce intestinal parasites in Timor-Leste: the WASH for WORMS research protocol. BMJ Open 5. 10.1136/bmjopen-2015-009293 PMC4710834 26719316

[40] Arndt MB, John-Stewart G, Richardson BA, Singa B, van Lieshout L, et al (2013) Impact of helminth diagnostic test performance on estimation of risk factors and outcomes in HIV-positive adults. PLoS One 8: e81915 doi: 10.1371/journal.pone.0081915 24324729 10.1371/journal.pone.0081915 PMC3852669

[41] Freeman MC, Chard AN, Nikolay B, Garn JV, Okoyo C, et al (2015) Associations between school- and household-level water, sanitation and hygiene conditions and soil-transmitted helminth infection among Kenyan school children. Parasit Vectors 8: 412 doi: 10.1186/s13071-015-1024-x 26248869 10.1186/s13071-015-1024-x PMC4528701

[42] Means AR, Burns P, Sinclair D, Walson JL (2016) Antihelminthics in helminth-endemic areas: effects on HIV disease progression. Cochrane Database Syst Rev 4: Cd006419 doi: 10.1002/14651858.CD006419.pub4 27075622 10.1002/14651858.CD006419.pub4 PMC4963621

[43] Campbell SJ, Nery SV, McCarthy JS, Gray DJ, Soares Magalhaes RJ, et al (2016) A Critical Appraisal of Control Strategies for Soil-Transmitted Helminths. Trends Parasitol 32: 97–107. doi: 10.1016/j.pt.2015.10.006 26795294 10.1016/j.pt.2015.10.006

[44] Means AR, Jacobson J, Mosher AW, Walson JL (2016) Integrated Healthcare Delivery: A Qualitative Research Approach to Identifying and Harmonizing Perspectives of Integrated Neglected Tropical Disease Programs. PLoS Negl Trop Dis 10: e0005085 doi: 10.1371/journal.pntd.0005085 27776127 10.1371/journal.pntd.0005085 PMC5077162

